# Hyperthermiphile biofilms of *Thermotoga neapolitana* on different materials and electrostimulated: SEM micrographs and chemical data of the glucose fermentation in electrochemical reactors

**DOI:** 10.1016/j.dib.2020.106403

**Published:** 2020-10-11

**Authors:** Gaetano Squadrito, Pierangela Cristiani, Giuliana d'Ippolito, Matteo Tucci, Nunzia Esercizio, Angela Sardo, Marco Vastano, Mariamichela Lanzilli, Angelo Fontana

**Affiliations:** a Institute of Biomolecular Chemsitry (ICB), National Research Council (CNR), Pozzuoli, NA, Italy; b Institute of Advanced Tecnologies for Energy (ITAE), National Research Council (CNR), Messina, Italy; c Water Research Institute (IRSA), National Research Council (CNR), Via Salaria km29, 300 00015 Monterotondo, Rome, Italy; d e-Bio Center, Department of Environmental Science and Policy, Università degli Studi di Milano, via Celoria 2, 20133 Milan, Italy; e RSE – Ricerca sul Sistema Energetico S.p.A., via Rubattino, 54, 20134 Milano, Italy

**Keywords:** *Thermotoga neapolitana*, Biofilm, SEM micorgraphs, Fixing samples, Bioelectrochemical system, Microbial electrostimulation, Carbon cloth, Glucose metabolism

## Abstract

Hyperthermophile bacteria were seldom investigated in bioelectrochemical systems although they allow more effective control of the inoculum in comparison with mesophilic bacteria. Biofilm formed in hyperthermophilic conditions (>60 °C) also rarely was documented (d'Ippolito et al., 2020; Belkin et al., 1986, Pysz et al., 2004).

Scanning Electron Microscopy (SEM) micrographs documenting biofilms formed by the Hyperthermophile bacterium *Thermotoga neapolitana* on different solid materials (ceramic carrier, stainless steel mesh, carbon felt, carbon paper, expanse graphite, and carbon cloth) are shown in this report. Also, micrographs of the biofilm formed on electrodes of carbon cloth under a dynamic polarization oscillating around ±1 V (±0.8 V and ±1.2 V) are reported.

Two procedures of sample preparation for SEM analyses are described and used: 1) a fast drying of samples, which is enough to underline the biofilm shape that covers solids, and 2) a chemical treating of the samples with glutaraldehyde, which better preserves the shape of bacterial cell components in the biofilm, although this treatment might cause the detachment of pieces of the biofilm.

The different effect of potentiostatic and potentiodynamic polarizations on the glucose metabolism of *T. neapolitana* has been screened and discussed in the associated article [1]. Here, data of Optical Densities (O.D.) of culture media are provided, indicating the presence or absence of bacteria growth in the bulk of the media. Data have been collected every 24 h from the differently polarized bioreactors. The electrodes set-up of small bioreactors is also illustrated.

Chemical data, optical data and SEM images, accordingly, document a retard in the glucose fermentation process due to a settlement of *T. Neapolitana* in a stationary phase. The polarization of electrodes can modify the stationary condition, inducing a possible change of the bacteria metabolism.

## Specifications Table

**Table:** 

Subject	Biotechnology
Specific subject area	Biofilm formed by hyperthermophile *Thermotoga neapolitana* on different materials and in bioelectrochemical systems
Type of data	Table Image Graph Figure
How data were acquired	Scanning Electron Microscopy (SEM), X Ray (EDX) probe and Optical microscopes, gas-chromatography, Nuclear Magnetic Resonance (NMR). Cyclic voltammetry (CV)
Data format	Raw, Analysed and graph
Parameters for data collection	*Thermotoga neapolitana* subsp. capnolactica (DSM33033) is investigated [2]. Biofilm growth on different materials: i) nonconductive (Al-Si ceramic carrier), ii) conductive (stainless steel AISI 304 and carbon-based), and iii) polarized (carbon cloth), in bioreactors is documented. Optical densities of the culture media, SEM micrographs of biofilm and bacteria cells are provided. Different acceleration voltages of 5 kV and 15 kV, increased to 20 kV are used for the EDX analysis. Two different fixing procedures for SEM samples, here described, are used.
Description of data collection	Tests were carried out at 80 °C in electrochemical bioreactors equipped with carbon cloth electrodes. The media of bioreactors was supplemented with 28 mM (0.5% wt/v) glucose and 0.4% (wt/v) yeast extract/tryptone for the bacteria growth. Carbon cloth electrodes were polarized imposing cyclic polarizations of ±0.8 V or ±1.2 V, with a scan rate of 0.5 mVs^−1^, operating in a two electrode configuration. Cyclic voltammetry (CV) at a scan rate of 50mVs^−1^ was performed on carbon cloth electrodes every 24 h. Electrodes were left in open circuit condition for 1 hour before the CV. Cell growth was determined as Optical Density (O.D.) at 540 nm wavelength with a spectrophotometer (Perkin Elmer Lambda 950).
Data source location	chemical analyses and optical observations: Institution: CNR-ICB City/Town/Region: Pozzuoli/Napoli Country: Italy SEM and EDX analyses: Institution: CNR-ITAE City/Town/Region: Messina Country: Italy SEM analyses: Institution: Ricerca sul Sistema Energetico - RSE City/Town/Region: Piacenza Country: Italy
Data accessibility	With the article
Related research article	G. d'Ippolito, G. Squadrito, M. Tucci, N. Esercizio, A. Sardo, M. Vastano, M. Lanzilli, A. Fontana, P. Cristiani. Electrostimulation of hyperthermophile *Thermotoga neapolitana* cultures. Bioresource Technology, DOI: 10.1016/j.biortech.2020.124078

## Value of the Data

• This article provides SEM images of *Thermotoga neapolitana* forming biofilms on different materials and polarized electrodes. Thermotogales were rarely documented on solid substrates. Different type of biofilm grew on polarized electrodes is documented. The reported information is useful to recognize different shapes of this bacterial species characterizing a stationary condition and a possible interaction with conductive electrodes.
• Two different procedures of sample preparation for SEM observations are described and used: a first based on drying the samples, to evidence biofilm, avoiding its detachment during the preparation; a second one based on chemical fixing by glutaraldehyde, useful to highlight the bacterial component shape, inside the biofilm.
• Biologist, Geochemists, and scientists of biotechnologies can take insight on *T. neapolitana* behavior and about the possibility to stress hyperthermophile microorganisms in bioelectrochemical systems for scientific and industrial applications.

## 1 Data Description

The hyperthermophile biofilm formed by *T. neapolitana* and its morphologies is documented here. Data referring to different polarization conditions for carbon cloth and different materials (conductive carbon and stainless steel, and insulating ceramic carrier) are provided. Such documentation of hyperthermophile biofilms is rarely available, with few exceptions [2,3], generally not concerning conductive substrates and polarized condition.

Table 1 summarize relevant characteristics of tested materials.

**Table 1:** Characteristics of materials for biofilm tests.

	Electrical Properties*	Porosity	Structure
Ceramic Al-Si porous carrier	> 10^10^ Ωcm	70%	Sintered ceramic grains
Stainless Steel net AISI 304	< 10^−10^ Ωcm		Plain net
Exp. Graphite foil	R_┴_ 650 μΩm; R_//_ 8 μΩm	n.a.	Compressed expanded graphite flakes
Carbon paper	< 1 Ωcm	70%	Carbon fibres covered with hydrophilic ink
Carbon Felt	R_┴_ 8 mΩcm^2^; R_//_ 5.4 mΩcm	n.a.	Not woven long carbon micro-fibres
Carbon cloth	1 Ωcm	n.a.	Plain woven long carbon micro-fibres

⁎ As reported in product datasheet for carbon materials; R_┴_ = trough plane resistance; R_//_ = in plane resistance

Two different procedures (Procedure A and Procedure B) for collecting and fixing biofilm samples were used. Operating in this way it was possible to maximize information from SEM observations, preserving the biofilm on the surface (by Procedure A) and having a high resolution in analyzing bacterial components shapes (by Procedure B). Micrographs of samples treated with Procedure A and B are both reported in the Figures. A higher acceleration voltage was used to better evidence bacteria in some SEM micrographs.

**Fig. 1:**
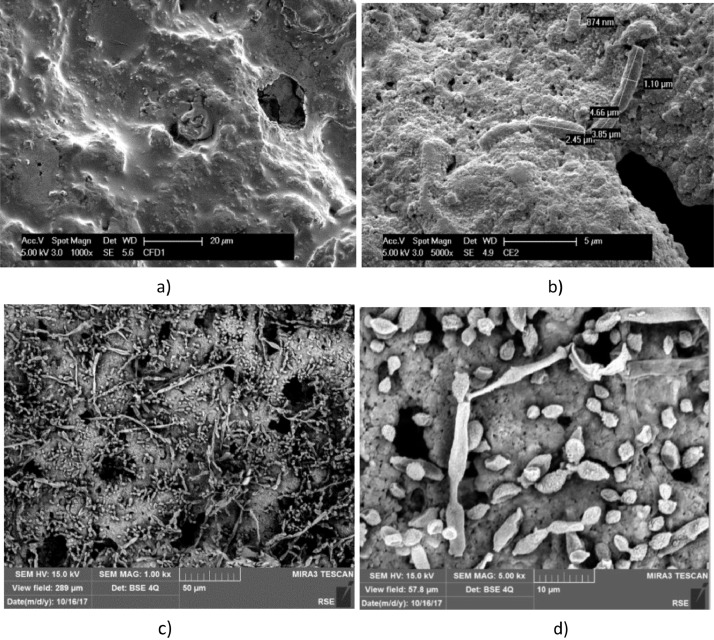
SEM micrographs, at different magnitudes, of a ceramic carrier exposed to *T. neapolitana* culture: for 6 days, dried with procedure A (a, b); exposed for 12 days, and fixed with procedure B (c, d). The dimension of some of the rod-shaped bacteria are reported in b).

**Fig. 2:**
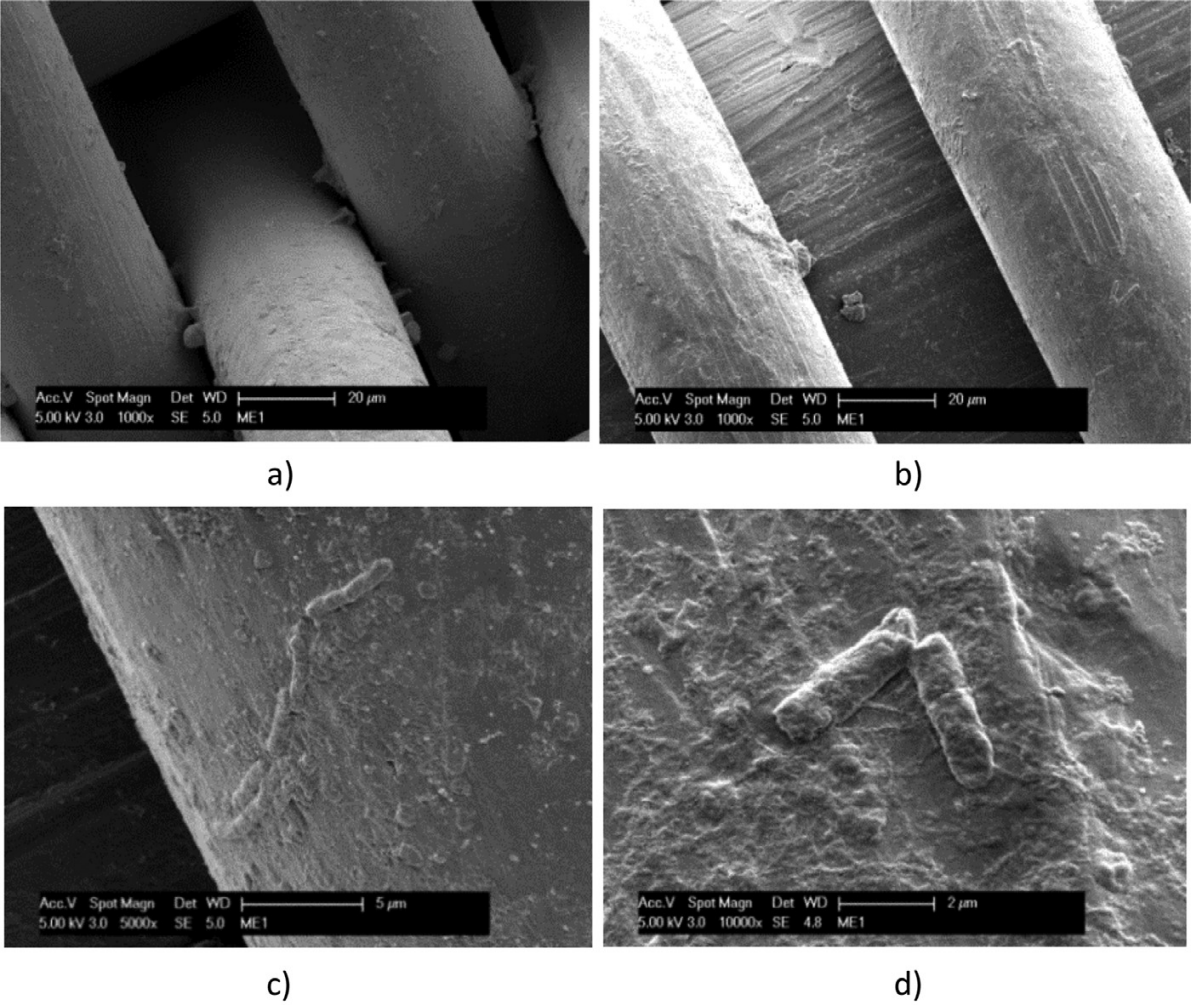
SEM micrographs of Stainless Steel operated in the *T. neapolitana* culture for 12 days: dried with Procedure A (a); fixed by Procedure B (b). Rod-shaped bacteria are highlighted in c) and d).

Fig. 1 shows the bacteria network developed inside the porosity of the ceramic carriers in less than a week and enriched in the second week. EDX data of ceramic carrier (showing a higher detection of carbon on biofilm than out of the biofilm) is reported in Supplementary File 1. Related EDX raw counts data are reported in Supplementary File 2. Fig. 2 shows much fewer bacteria attached to the high conductive and plate surface of Stainless Steel for two weeks. The bacterial cells are clearly rod-shaped in this case.

**Fig. 3:**
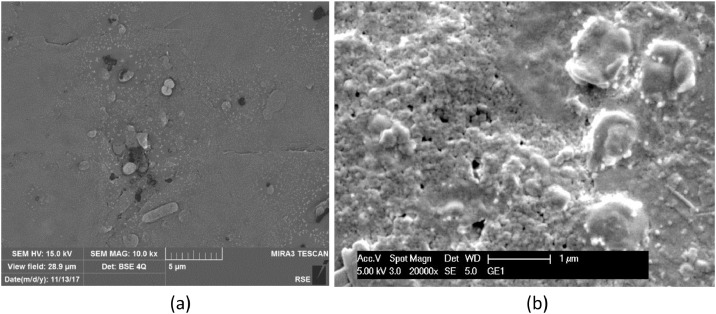
SEM micrographs of graphite foil operated in the *T. neapolitana* culture for 12 days: dried with Procedure A (a); fixed with Procedure B (b). a) micrograph achieved at low acceleration voltage and b) at a high acceleration voltage.

**Fig. 4:**
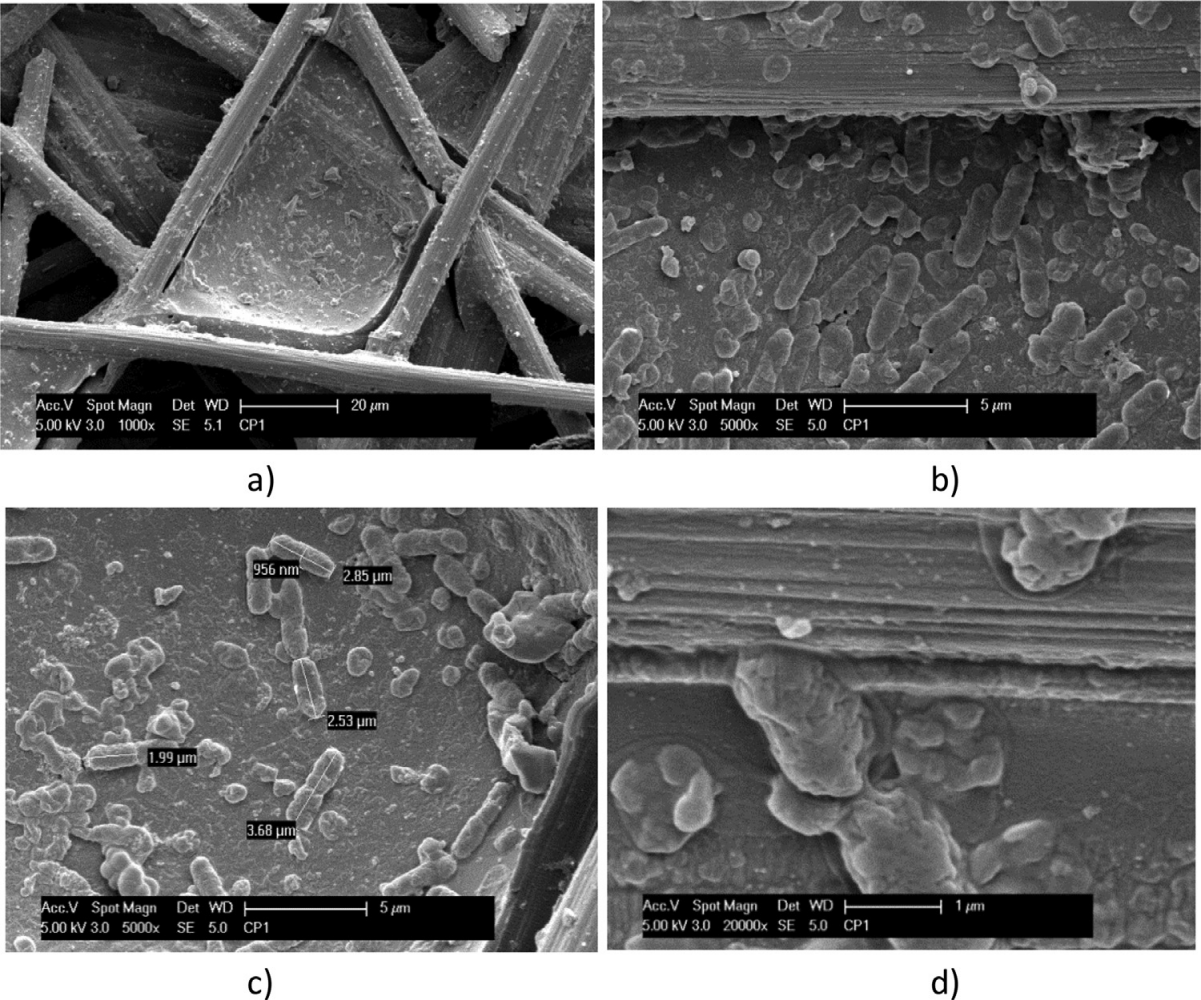
SEM micrographs, at different magnitudes, of Carbon paper exposed to *T. neapolitana* for 12 days: samples dried by Procedure A (a–c); Sample fixed by Procedure B (d). Length of rod-shaped detected bacteria are reported in c). The toga covering bacteria and attached to the support is evidenced in d).

**Fig. 5:**
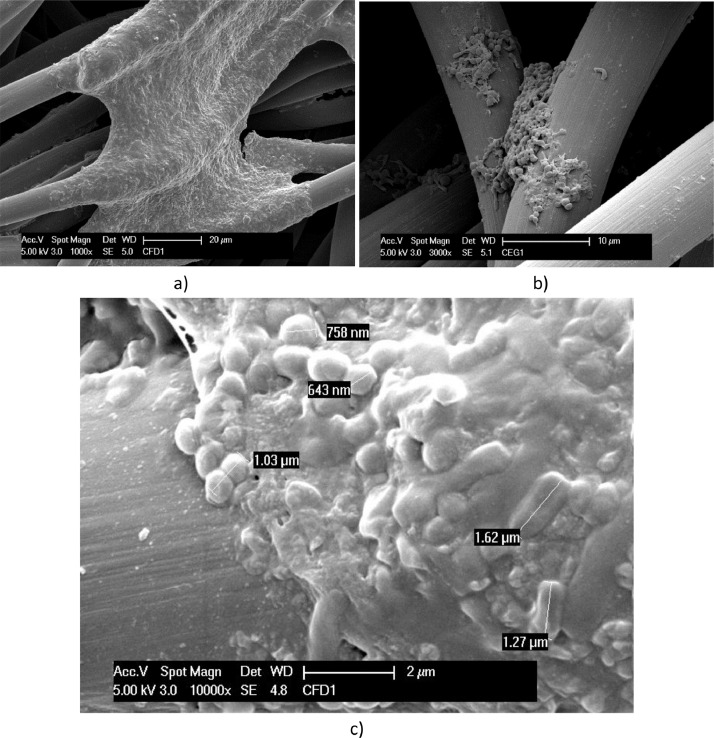
SEM micrographs, at different magnitudes, of Carbon felt exposed to *T. neapolitana* for 12 days: dried by Procedure A (a); fixed by procedure B (b and c). Coccoid and rod-shaped bacteria are highlighted and measured in c).

**Fig. 6:**
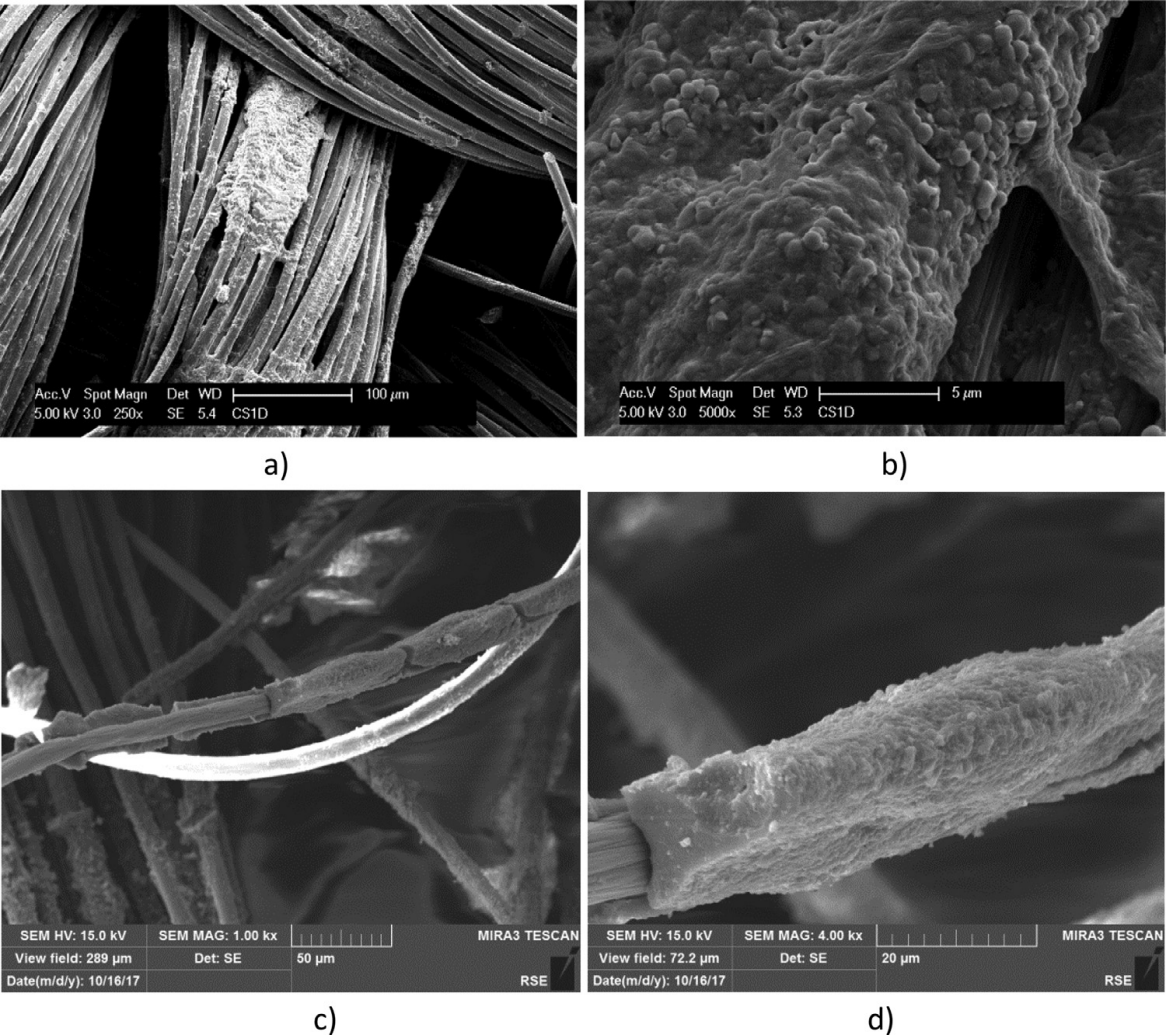
SEM micrographs, at different magnitudes, of Carbon cloth exposed to *T. neapolitana* for 12 days and dried by Procedure A (a - d). Biofilm covering a single carbon cloth fibre is evidenced in c) and d).

**Fig. 7:**
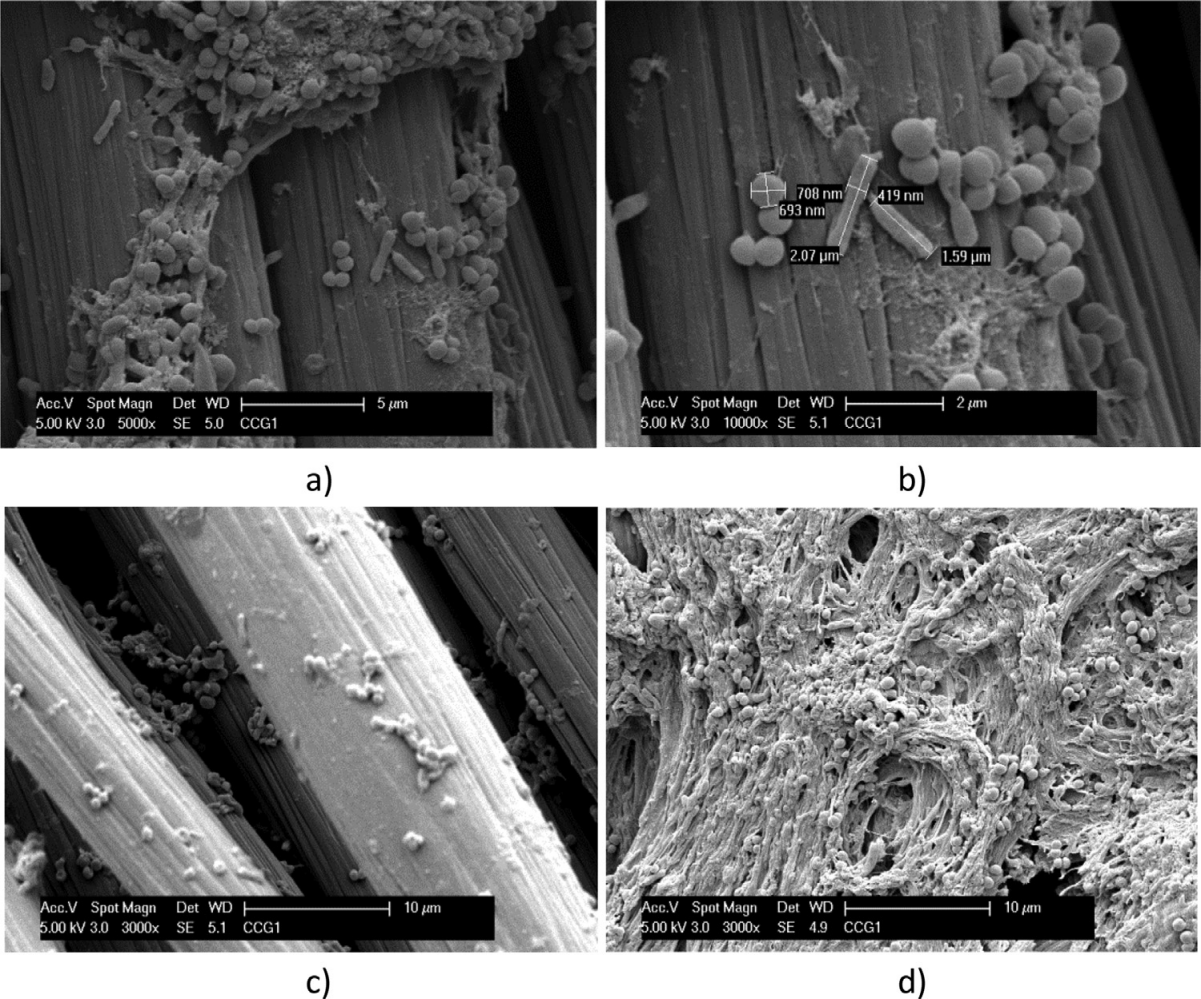
SEM micrographs at different magnitude of Carbon cloth exposed to T. neapolitana for 12 days and fixed by Procedure B (a – d). Coccoid and rod forms of bacteria attached to a carbon cloth fiber are evidenced and measured in b). A dense and filamentous network of bacteria is evidenced in d), which element composition is analyzed by EDX spectra reported in e).

Figs. 3–7 show biofilm formed on conductive carbon materials described in Table 1. Rod-shaped bacteria on a plate graphite foil, like on Stainless Steel, are shown in Fig. 3. Fig. 4 highlights a more rich community of rod-shaped bacteria settled on carbon paper. Rod-shaped bacteria are visible also on carbon felt in Fig. 5, but mixed with Coccoid forms. EDX data of Carbon Felt fully covered by biofilm, dried and Gold plated is reported in Supplementary File 1. Related EDX raw data are reported in Supplementary File 2.

A biofilm richer of bacteria in a coccoid form is evidenced on carbon cloth in Figs. 6 and 7. Carbon cloth was selected for testing the effect of a dynamic electrostimulation, that was carried out for three days in small single chamber electrochemical reactors.

The effect of static and potentiodynamic electrostimulations of *T.neapolitana* cultures, under different electrochemical conditions, is discussed in the associated paper [1]. Bacteria interaction with carbon cloth electrodes was evidenced in that work by cyclic voltammetry and differences in the trend of glucose fermentation. Tests were carried out in different types of bioreactors (large and small bioreactors [1]).

**Fig. 8:**
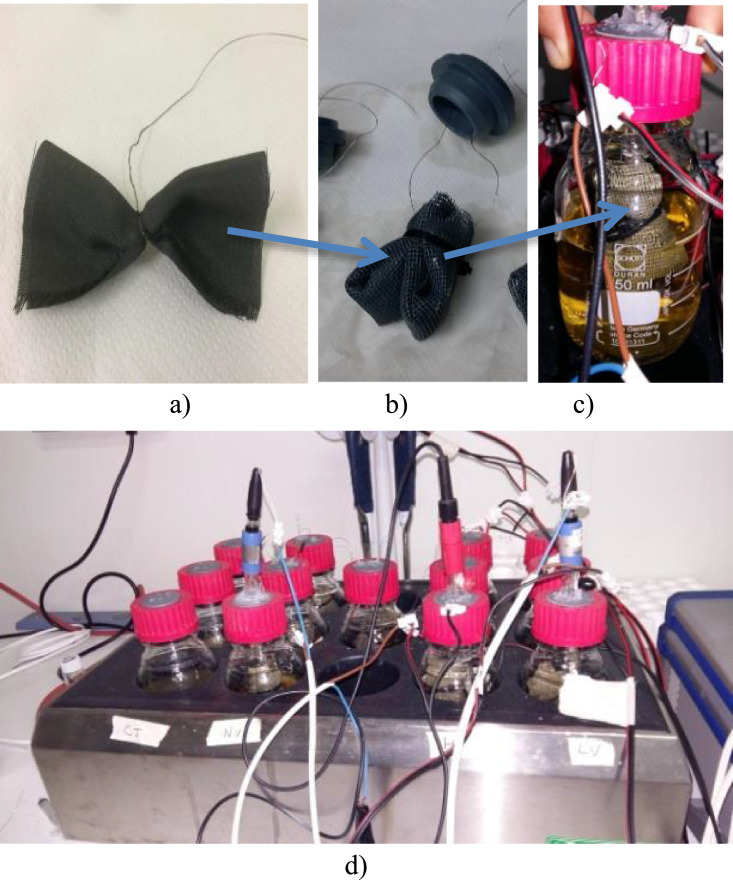
a-c: set-up of carbon cloth electrodes and bioreactors. Carbon cloth (10 × 10 cm) was clenched to a titanium wire (collector) (a), then insulated in a plastic net and coupled with another identical electrode (b), and finally immersed in a small bioreactor (c). Bioreactors altogether were kept at 80 °C in a thermostat (d). One of the triplicates for each polarization condition is equipped with an additional reference electrode (Ag/AgCl 3 M) to measure the potential of carbon cloth electrodes. Two of the tested bioreactors (triplicate, 15 in total) are missing in the photo.

**Fig. 9:**
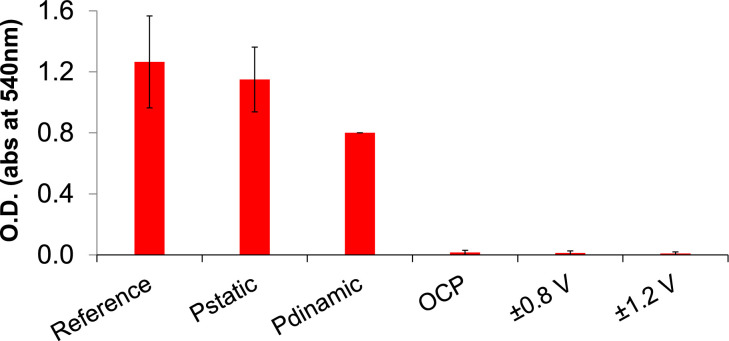
Histogram of O.D. data (average with error bars) of all tested bioreactors. Reference includes control tests (without electrodes) carried out in small and large bioreactors altogether; Pstatic and Pdinamic are potentiostatic and potentiodynamic polarization, respectively, carried out in the large bioreactor. The other tests (open circuit potential OCP, and potentiodynamic polarization between ±0.8 V, and ±1.2 V) are carried out in small bioreactors [1]. Raw data are reported in the Supplementary File 3.

**Fig. 10:**
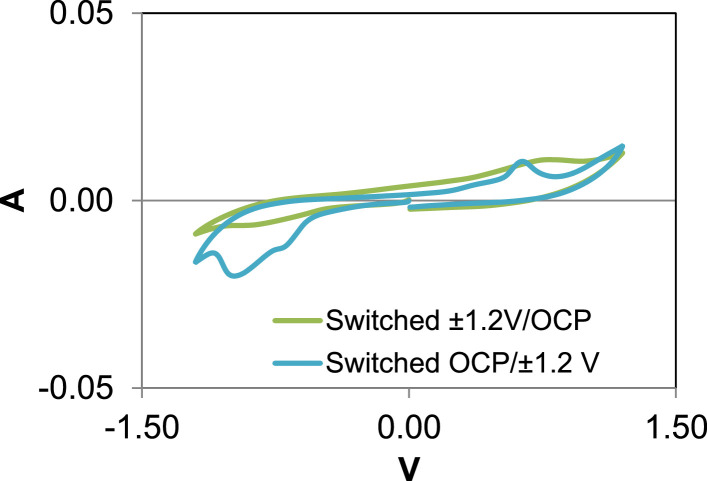
Cyclic voltammetry carried out after three days of test on electrodes of the two bioreactors which polarization was switched at the beginning of the second day from ±1.2 V to OCP and from OCP to ±1.2 V, respectively.

Details of the set-up for carbon cloth electrodes of small bioreactors (thermostated at 80 °C) are illustrated here in Fig. 8. O.D. data after 24 h of testing with small and large bioreactors, showing abundancy or scarceness of bacteria in the bulk media of each bioreactor (as described in [1]) are shown in Fig. 9. Raw data of O.D. and of chemical analyses sampled after 24 h testing both from large and small bioreactors media are reported in the Supplementary File 3. Also, cyclic CVs of electrodes which polarization condition was reversed the second day of the test: one (Reactor n. 9) from open circuit condition (OCP) to ±1.2 V, and the other (reactor n. 6) from ±1.2 V to OCP, not replicated, are shown in the graphic of Fig. 10.

**Fig. 11:**
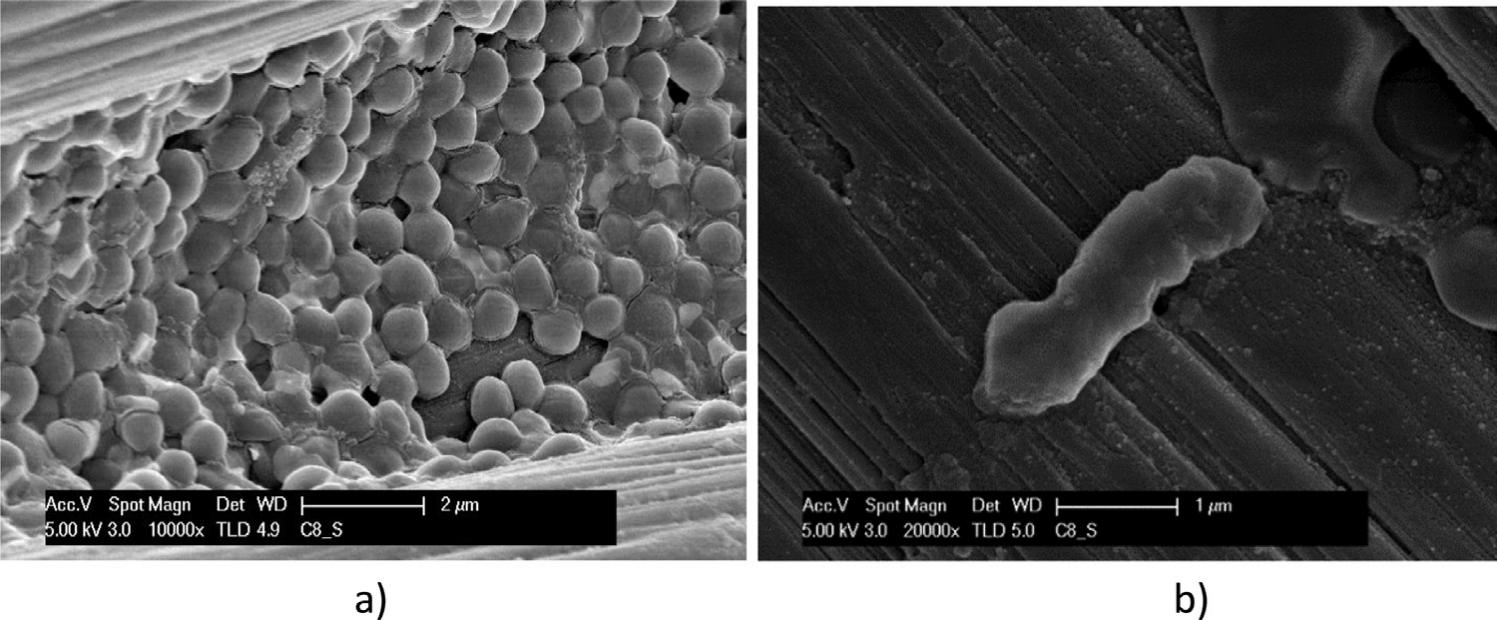
SEM micrographs, at different magnitudes, of carbon cloth electrodes operated in OCP condition for 3 days: dried with Procedure A (a); fixed with Procedure B (b).

**Fig. 12:**
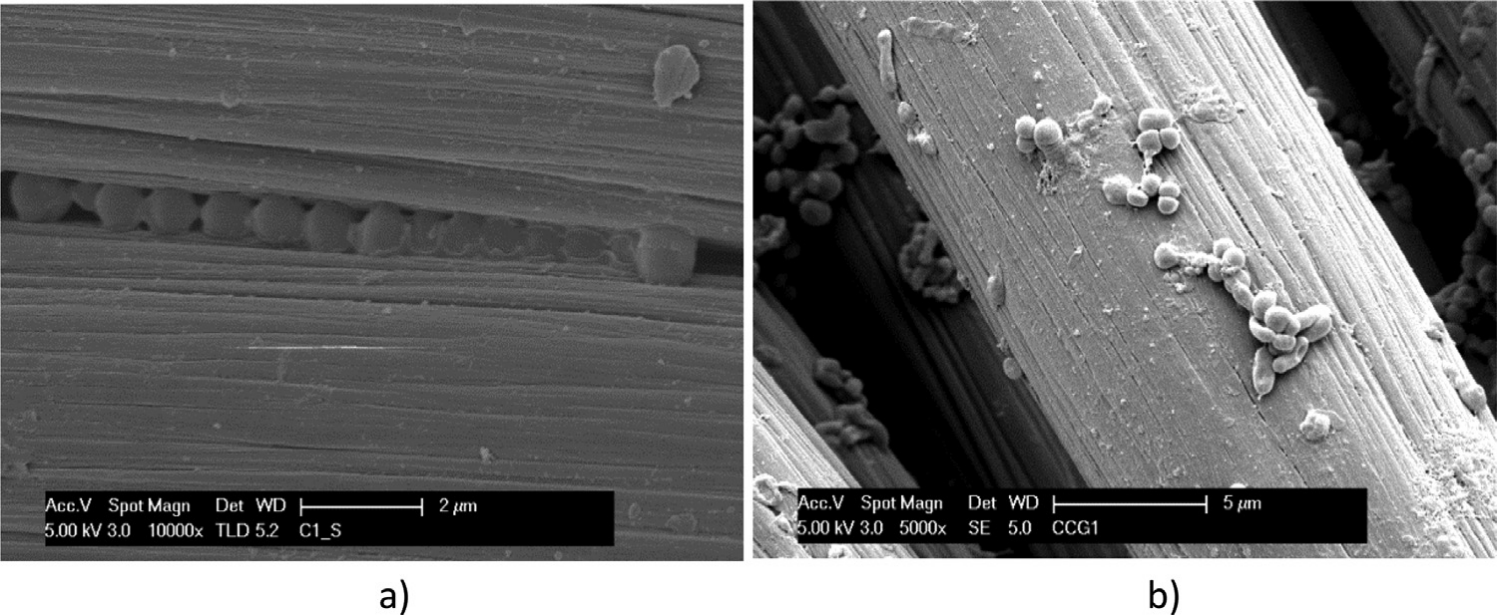
SEM micrographs, at different magnitudes, of carbon cloth, operated cycling ± 0.8 V between electrodes for 3 days: dried with Procedure A (a); fixed with Procedure B (b).

**Fig. 13:**
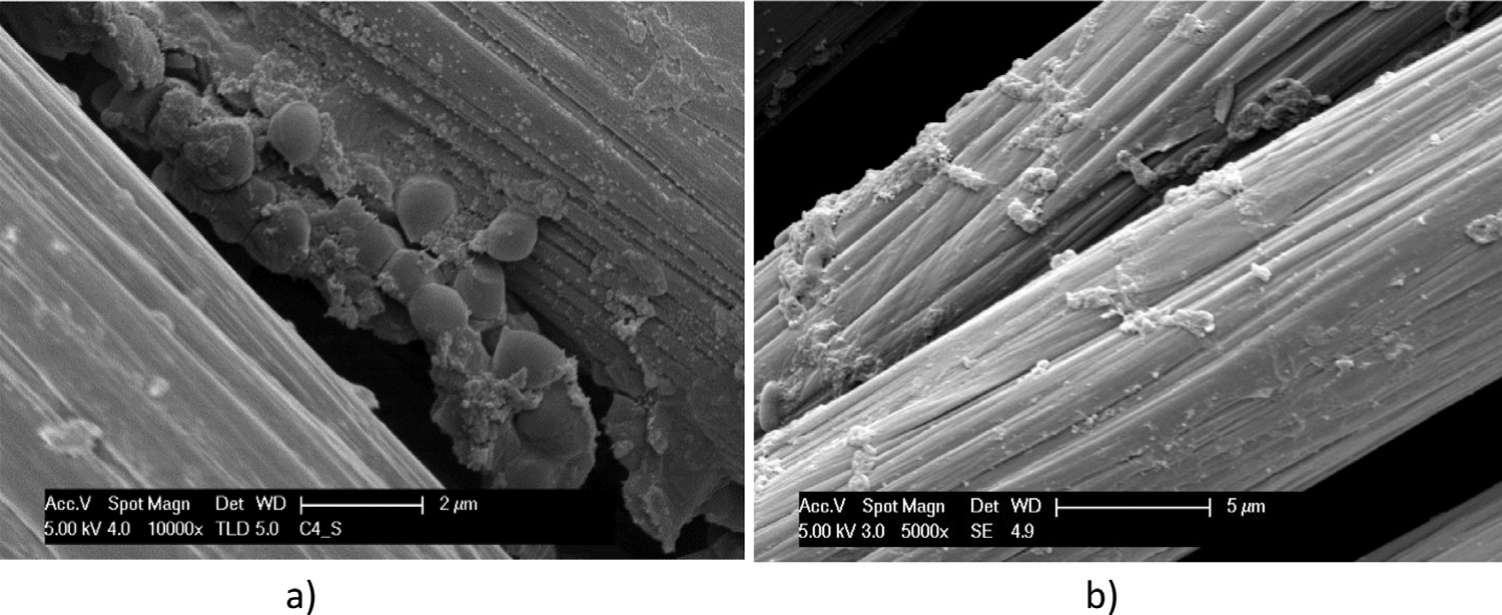
SEM micrographs, at different magnitudes, of carbon cloth, operated cycling ± 1.2 V between electrodes for 3 days: dried with Procedure A (a); fixed with Procedure B (b).

Biofilm formed on the surface of carbon cloth under different conditions of polarization after 72 h testing is documented in the SEM micrographs of Figs. 11 and 15. Coccoid (more than rod) bacteria forms are evidenced on electrodes of carbon cloth kept in OCP condition during the test, as shown in Fig. 11. Figs. 12 and 13 show the biofilm found on carbon cloth operated cycling continuously ± 0.8 V and ± 1.2 V, respectively.

**Fig. 14:**
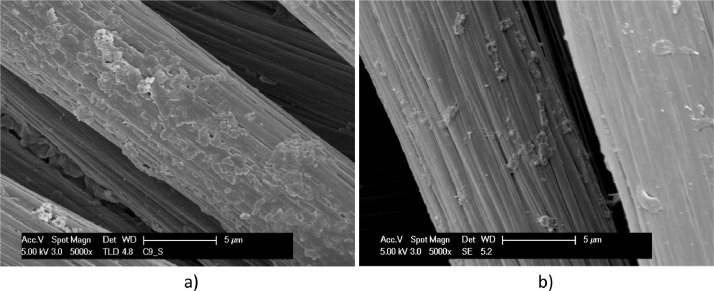
SEM micrographs of carbon cloth operated cycling ± 1.2 V between electrodes for 1 day, then kept in open circuit for 2 days (±1.2 V/OCP): sample dried by Procedure A (a); fixed with Procedure B (b).

**Fig. 15:**
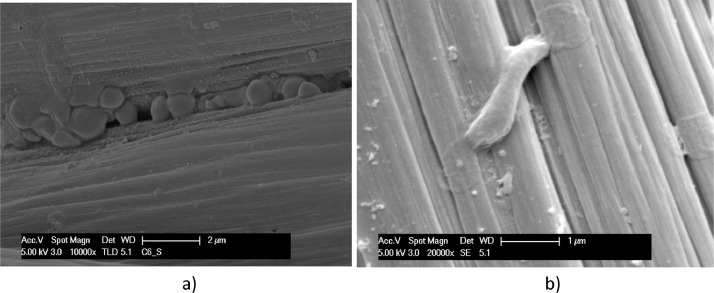
SEM micrographs of carbon cloth, operated in open-circuit for 1 day, then cycling ± 1.2 V between electrodes for 2 days (OCP/±1.2 V): sample dried by Procedure A (a); fixed with Procedure B (b).

Figs. 14 and 15 show the biofilm on electrodes in switched reactors, which polarization was modified during the test (after the first day) from a condition to another (±1.2 V/OCP and OCP/±1.2 V, respectively).

Supplementary File 3 reports raw data of analyzed chemical components of glucose fermentation (concentration of glucose, acetic and lactic acids, hydrogen), as well as raw data of O.D., for 24 h testing in both large and small bioreactors.

## 2 Experimental Design, Materials and Methods

*Thermotoga neapolitana* subsp. capnolactica (DSM33033), a lab strain derived from *T. neapolitana* DSMZ 4359T [2] was investigated. The set-up of electrochemical reactors, the bacteria culture, and media are described in the associated reference [1].

### 2.1 Electrode set-up

A carbon cloth piece of 10 × 10 cm was used for setting up electrodes of small reactors (315 mL) [1]. The carbon cloth piece was wrapped-around and thigh fixed to a titanium wire, as shown in Fig. 8a, and then wrapped again with a plastic net (Fig. 8b) so to avoid short-circuits when pressed close to the opposite electrode. Electrodes were dimensioned to geometrically occupy almost 1/5 of the liquid volume when immersed almost completely in the reactor (Fig. 8c).

### 2.2 Materials for biofilm tests

The tested materials were: conductive carbon cloth (SAATI, Legnano, Italy), carbon paper (SpectraCarb), carbon felt (Freudenberg), stainless steel (AISI 304) mesh, expanded graphite (SGL Carbon). A porous, non-conductive Al-Si ceramic carrier was also investigated. The same Carbon Cloth (SAATI, Legnano, Italy) was the conductive material used both for electrostimulation tests and for biofilm tests. The same bottles (DURAN) of 250 ml with a liquid volume of 150 ml were used for the biofilm tests and for the electrostimulation tests. The materials were inserted in the medium before the autoclave step. The biofilm tests lasted 6 and 12 days and were performed without stirring.

The characteristics of tested materials are reported in Table 1.

### 2.3 Sampling procedures for SEM analysis

After two weeks of cultivation experiment, the supports immersed in the small reactors were recovered and gently washed with an isotonic solution (NaCl 10 gL^−1^). Each material was treated in two different ways, as follows.

Sampling Procedure A: The materials were dried 30 minutes in the oven at 80 °C and stored at 4 °C until SEM analysis. The dried samples were dried a second time for 1 hour at 80 °C, mounted on the sample holder, and golden using a cold magnetron sputtering.

Sampling Procedure B: the samples were stored in a 2% glutaraldehyde solution (solution in 10gL^−1^NaCl), and stored at 4 °C until SEM analysis. Before, SEM observation they have been washed in alcoholic solution (25, 50, 100%) with steps of 2 minutes, then dried at 50 °C and 80 °C (2 h each), cut and mounted on the SEM sample holder, then covered with gold by cold magnetron sputtering.

SEM analysis was carried out by two institutions (CNR and RSE): with an FEI-XL30-SFEG (CNR, at Messina, Italy) and with a Mirai3-Tescan (RSE, at Piacenza, Italy), using acceleration voltages in the range 5–15 kV.

A higher acceleration (20 kV) was used for the EDX analysis. The bacteria settlement was analyzed on both the faces of the flat samples and a significant difference attributable to the gravity effect was not found in any cases.

### 2.4 Optical density measurements

Cell growth was determined as Optical Density (O.D.) at 540 nm wavelength with a spectrophotometer (Perkin Elmer Lambda 950). The OD_540nm_ value of each culture was corrected for the background absorbance of the supernatant after cell harvesting (10 min, 6000 g).

## 5 Supplementary Files

Supplementary Files 1 and 2 report information and raw data of EDX analyses.

Supplementary File 3 reports raw chemical data of O.D. and concentration of components of glucose fermentation (glucose, acetic and lactic acids, and hydrogen) of media sampled from both small and large bioreactors after 24 h of testing [1]; yields of the glucose fermentation are also reported in the file.

## Ethics Statement

Not applicable.

## Declaration of Competing Interest

The authors declare that they have no known competing financial interests or personal relationships which have, or could be perceived to have, influenced the work reported in this article.

## Appendix Supplementary materials

Image, application 1

Image, application 2

Image, application 3
