# Supplementary material for: Hyperthermiphile biofilms of Thermotoga neapolitana on different materials and electrostimulated: SEM micrographs and chemical data of the glucose fermentation in electrochemical reactors
Source: Data Brief. 2020 Oct 11;33:106403. doi: 10.1016/j.dib.2020.106403 (PMC7581883; doi:10.1016/j.dib.2020.106403)

**EDX Figure 1**: **Ceramic carrier covered by biofilm**

EDAX ZAF Quantification (Standardless)

Element Normalized

SEC Table : Default

Elem Wt % At % K-Ratio Z A F

-------------------------------------------------------------

C K 30.50 44.40 0.0576 1.0408 0.1813 1.0003

O K 31.02 33.90 0.0723 1.0233 0.2276 1.0003

NaK 1.99 1.51 0.0086 0.9578 0.4479 1.0037

MgK 5.34 3.84 0.0309 0.9819 0.5860 1.0054

AlK 10.55 6.83 0.0672 0.9531 0.6649 1.0048

SiK 12.85 8.00 0.0855 0.9809 0.6778 1.0004

AuM 5.57 0.49 0.0395 0.6893 1.0289 1.0001

ClK 1.12 0.55 0.0083 0.9237 0.8056 1.0007

K K 0.45 0.20 0.0037 0.9301 0.8960 1.0009

CaK 0.62 0.27 0.0054 0.9521 0.9262 1.0000

Total 100.00 100.00

Element Net Inte. Backgrd Inte. Error P/B

-------------------------------------------------

C K 13.21 0.94 3.36 14.07

O K 43.92 0.94 1.76 46.76

NaK 7.38 1.55 5.01 4.76

MgK 27.25 1.88 2.33 14.51

AlK 58.63 1.88 1.54 31.22

SiK 69.64 2.11 1.41 32.96

AuM 8.82 2.17 4.70 4.07

ClK 5.50 1.84 6.29 2.99

K K 2.09 1.58 12.53 1.32

CaK 2.77 1.54 9.98 1.80

kV: 20.00 Tilt: 0.00 Take-off: 34.85 Tc: 100.0

Det Type:SUTW, Sapphire Res: 128.41


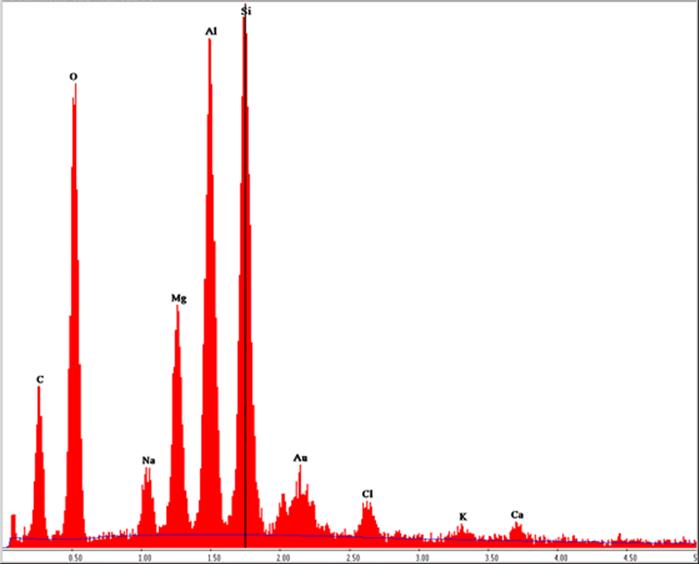


**EDX Figure 1: Ceramic carrier without visible biofilm (Fracture)**

EDAX ZAF Quantification (Standardless)

Element Normalized

SEC Table : Default

Elem Wt % At % K-Ratio Z A F

-------------------------------------------------------------

C K 7.44 13.30 0.0104 1.0608 0.1315 1.0004

O K 35.14 47.16 0.1012 1.0430 0.2760 1.0005

NaK 0.94 0.88 0.0041 0.9760 0.4422 1.0055

MgK 8.21 7.25 0.0487 1.0005 0.5875 1.0079

AlK 16.57 13.18 0.1025 0.9710 0.6328 1.0066

SiK 21.21 16.21 0.1283 0.9993 0.6052 1.0003

AuM 8.60 0.94 0.0535 0.7047 0.8823 1.0001

ClK 0.72 0.43 0.0048 0.9447 0.7144 1.0006

K K 0.38 0.21 0.0030 0.9496 0.8331 1.0009

CaK 0.79 0.42 0.0067 0.9717 0.8753 1.0000

Total 100.00 100.00

Element Net Inte. Backgrd Inte. Error P/B

-------------------------------------------------

C K 0.62 0.35 17.31 1.77

O K 16.03 0.21 2.37 76.21

NaK 0.92 0.35 12.95 2.62

MgK 11.17 0.42 2.90 26.56

AlK 23.29 0.49 1.98 47.46

SiK 27.19 0.62 1.84 43.71

AuM 3.11 0.70 6.39 4.44

ClK 0.83 0.70 16.81 1.19

K K 0.44 0.67 28.57 0.65

CaK 0.88 0.63 15.50 1.40

kV: 20.00 Tilt: 0.00 Take-off: 35.00 Tc: 100.0

Det Type:SUTW, Sapphire Res: 128.41


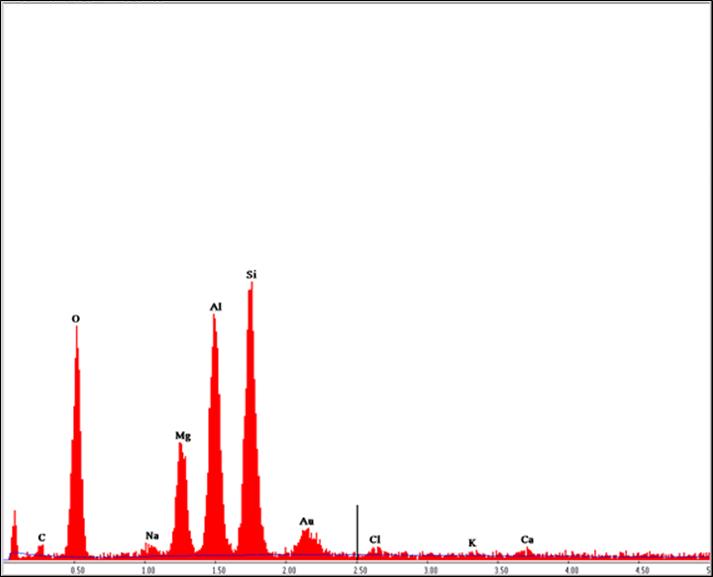


**EDX Figure 5: Carbon Felt** **fully covered by biofilm, dried and Gold plated**

EDAX ZAF Quantification (Standardless)

Element Normalized

SEC Table : Default

Elem Wt % At % K-Ratio Z A F

-------------------------------------------------------------

C K 63.08 79.20 0.1466 1.0340 0.2247 1.0001

O K 10.11 9.53 0.0161 1.0167 0.1568 1.0002

NaK 9.08 5.96 0.0424 0.9517 0.4906 1.0005

AuM 6.27 0.48 0.0566 0.6842 1.3181 1.0009

ClK 10.52 4.47 0.0897 0.9168 0.9300 1.0008

K K 0.94 0.36 0.0080 0.9235 0.9211 1.0000

Total 100.00 100.00

Element Net Inte. Backgrd Inte. Error P/B

-------------------------------------------------

C K 25.29 0.48 2.85 53.08

O K 7.34 0.44 5.50 16.82

NaK 27.39 0.95 2.78 28.75

AuM 9.47 3.49 6.04 2.71

ClK 44.36 3.27 2.27 13.55

K K 3.33 0.67 9.14 4.94

kV: 20.00 Tilt: 0.00 Take-off: 35.14 Tc: 100.0

Det Type:SUTW, Sapphire Res: 128.41


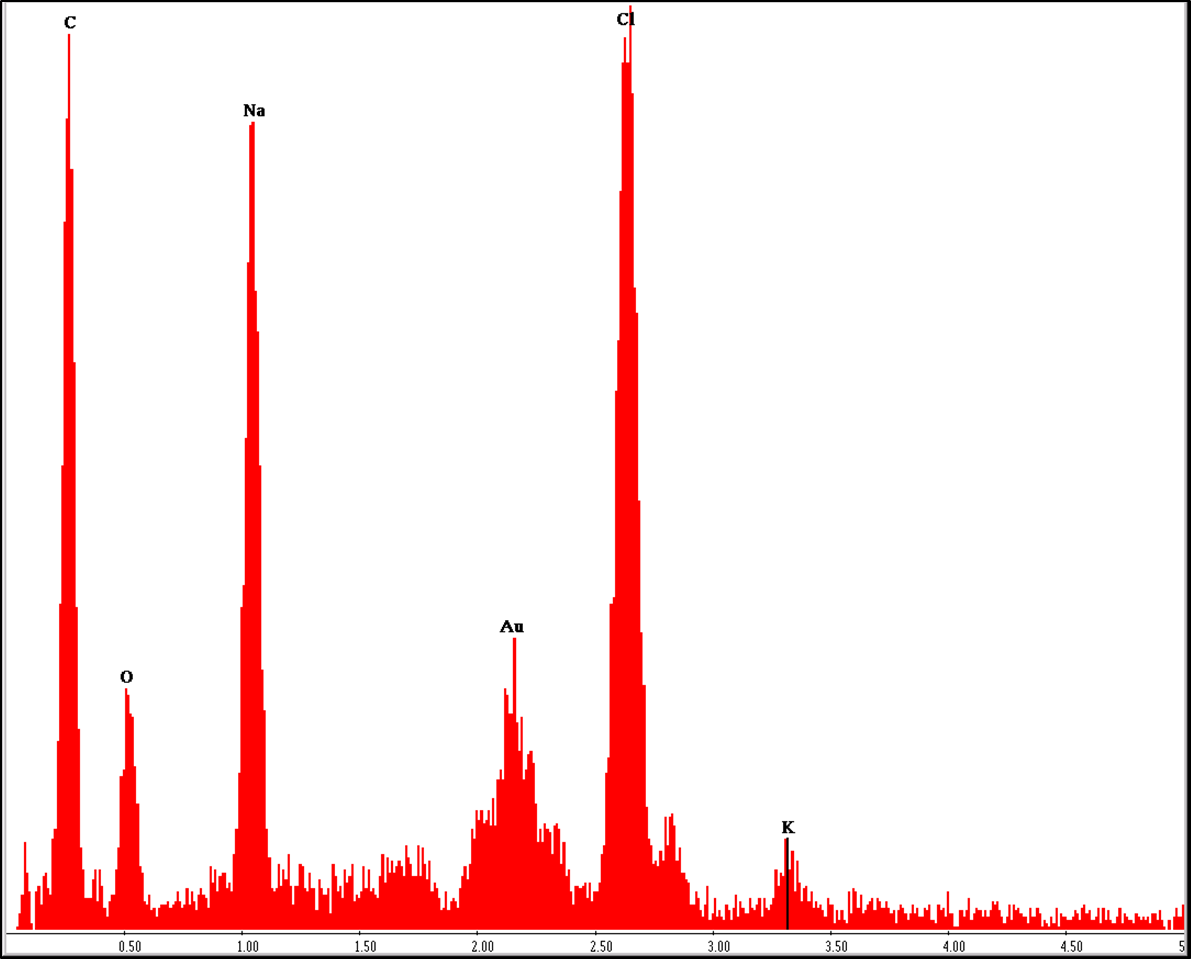

Supplement: Supplementary file 1 [file mmc1.docx]
